# The role of Th1 and Th17 cells in glomerulonephritis

**DOI:** 10.12860/jnp.2015.07

**Published:** 2015-03-19

**Authors:** Fatemeh Azadegan-Dehkordi, Nader Bagheri, Hedayatollah Shirzad, Mahmoud Rafieian-Kopaei

**Affiliations:** ^1^Cellular and Molecular Research Center, Shahrekord University of Medical Sciences, Shahrekord, Iran; ^2^Department of Immunology, School of Public Health, Tehran University of Medical Sciences, Tehran, Iran; ^3^Medical Plants Research Center, Shahrekord University of Medical Sciences, Shahrekord, Iran

**Keywords:** Renal injury, Glomerulonephritis, Chronic kidney disease, Autoimmune disease

## Abstract

*Context:* T helper (Th) cells as an important part of the immune is responsible for elimination of invading pathogens. But, if Th cell responses are not regulated effectively, the autoimmune diseases might develop. The Th17 subset usually produces interleukin-17A which in experimental models of organ-specific autoimmune inflammation is very important.

*Evidence Acquisitions:* Directory of open access journals (DOAJ), Google Scholar, Embase, Scopus, PubMed and Web of Science have been searched.

*Results:* Fifty-six articles were found and searched. In the present review article, we tried to summarize the recently published data about characteristics and role of Th1 and Th17 cells and discuss in detail, the potential role of these T helpers immune responses in renal inflammation and renal injury, focusing on glomerulonephritis. Published papers in animal and human studies indicated that autoimmune diseases such as rheumatoid arthritis and multiple sclerosis, classically believed to be Th1-mediated, are mainly derived from a Th17 immune response. Identification of the Th17 subgroup has explained seemingly paradoxical observations and improved our understanding of immune-mediated inflammatory responses.

* Conclusions:* Secretion of IL-17A, as well as IL-17F, IL-21, IL-22, suggests that Th17 subset may play a crucial role as a pleiotropic pro-inflammatory Th subset. There is experimental evidence to support the notion that Th1 and Th17 cells contribute to kidney injury in renal inflammatory diseases like glomerulonephritis.

**Implication for health policy/practice/research/medical education:**

Identification of the Th17 subgroup has explained paradoxical observations and improved our understanding of immune-mediated inflammatory responses. Secretion of IL-17A, as well as IL-17F, IL-21, IL-22, suggests that Th17 subset may play a crucial role as a pleiotropic pro-inflammatory Th subset. There is experimental evidence to support the notion that Th1 and Th17 cells contribute to kidney injury in renal inflammatory diseases like glomerulonephritis.

##  Context

Renal failure is considered as the most serious outcome of the chronic kidney disease (CKD) in which the symptoms are mainly caused by complications of decreased kidney function. In this situation when the symptoms are severe and the renal function permanently failed, they might be treated only by transplantation or dialysis. The kidney failure in this situation is known as end-stage renal disease (1-5).

## 2. Evidence acquisition

We searched various electronic databases, including Directory of open access journals (DOAJ), Google Scholar, Embase, Scopus, PubMed and Web of Science. We searched keywords of renal injury, glomerulonephritis, CKD and autoimmune disease. For this review, fifty-six articles were found and searched.

## 3. Results

The kidneys are frequently targeted by local manifestations of systemic autoimmunity or pathogenic immune responses such as renal autoantigens. Animal and human studies have recently uncovered several underlying mechanisms that might be used to explain the previously enigmatic immunopathology of many renal diseases. These mechanisms include kidney-specific damage-associated molecular patterns that cause crosstalk between renal dendritic cells and T cells, development of kidney-targeting autoantibodies, sterile inflammation, and molecular mimicry with microbial pathogens. Infectious and cardiovascular diseases are the most important reasons for the high mortality and morbidity associated with CKD and end-stage renal disease (ESRD). It is estimated that more than 15% of the adult subjects have some degree of CKD, and that dialysis which is applied on about 0.1% of the population consumes about 2% of total health expenditure in most developed countries (6). The T cells are involved in a lot of immune-mediated diseases, including the ones affecting kidney (7,8). They act as “helpers” for B cells which produce autoantibodies against kidneys, for example in Goodpasture syndrome, a form of type 2 hypersensitivity, or antibodies which form immune complexes, that upon glomerular deposition induce type 3 hypersensitivities, also known as immune complex glomerulonephritis (GN). However, there is a report suggesting a role for the fourth “cellular” delayed type of hypersensitivity in human glomerulonephritis (9).

### 3.1. Differentiation and function of Th1 and Th17 cells

CD4^+^ T-helper cells have been shown to play a crucial role in the regulation of the adaptive immune response. Activation of CD4^+^ T-helper cells is critical for the control and elimination of invading pathogens, however, autoimmune disease might arise if CD4^+^ T cells become activated in response to self-antigens. A key mechanism used by the immune system to prevent autoimmunity is to precisely regulate the differentiation and activation of CD4^+^ T-helper cells (10). Based on the cytokine secretion and expression of specific transcription factors, the CD4^+^ T cells are classified into four major sub-groups: Th1, Th2, Th17, and regulatory T cells (Tregs), although further T-helper cell lineages might exist (11). It has been suggested that CD4^+^ T cells might be subgroup into two independent subsets, called Th1 and Th2 cells (12). Th1 cells mainly produce interferon-γ (IFN-γ), which is crucial for macrophage activation, and are predominantly involved in the clearance of intracellular pathogens. Th2 cells are, in contrast, characterized by the production of IL-4, IL-5, and IL-13 and are critical for eosinophil recruitment, IgE production, and participate in the elimination of extracellular pathogens. Interleukin-12, is critical for Th1 differentiation, however, IL-4 has the major role in Th2 differentiation. Data from experimental and human experimental models have provided strong evidence that, Th1 cells might also be involved in cell-mediated autoimmune disease (for example, multiple sclerosis, Crohn’s disease, rheumatoid arthritis, and crescentic glomerulonephritis), whereas Th2 cells are associated with allergic diseases. Th17 cells differ from Th1 and Th2 cells by cytokines that drive their differentiation and also by their cytokine expression profile. Under the influence of IL-6, transforming growth factor-β (TGF-β), IL-1, and IL-21, the key transcription factors are known to induced and mediate Th17 cell differentiation in humans and animals (11). Remarkably, recent studies have indicated that there might be also TGF-β-independent pathways for differentiation of Th17 cells (13). Interleukin-23 is also essential for expansion and stabilization of the Th17 cell population. Th17 cells are implicated in organ-specific autoimmune inflammation and their roles in glomerulonephritis. The discovery of Th17 cells in mice came from the experiments that documented the effects of IL-12 and IL-23 in experimental murine models of rheumatoid arthritis, inflammatory bowel disease and multiple sclerosis (14,15). In all three models of autoimmune disease, IL-23 have played an important role whereby IL-23-deficient have been completely protected from disease. Interleukin-12 and IL-23 are heterodimeric cytokines of the same family and share the same p40 subunit with different second subunits, p35 and p19, respectively (16).

It was previously believed that IL-12 was the key cytokine in inflammatory diseases as neutralizing antibodies to p40 ameliorated disease in experimental autoimmune encephalomyelitis (EAE) (17). Interleukin-12 had been known to direct Th1– IFN-γ responses (18,19), and inflammatory diseases were known to cause by an unregulated Th1 response. However, it was unexpectedly observed that mice deficient in IFN-γ or its receptor were not protected from EAE (20,21). Shortly after report of these paradoxical observations, the IL-23 p19 subunit was discovered (16) and as named, IL-23 which is now regarded as the key cytokine in the pathogenesis of mouse inflammatory bowel disease and collagen induced arthritis (CIA). Experimental trials in EAE have shown that IL-23 is responsible for driving the development and expansion of the distinct Th17 subset that produces tumor necrosis factor (TNF)-α, IL-6, IL-17A and IL-17F (22). Furthermore, the pathogenicity of IL-23-driven Th17 cells has been proven by transfer of these cells into recipient animals that consequently developed severe clinical signs of EAE (22). Neutralization of IL-17A has been shown to correlate with protection from EAE (22). IL-17- deficient mouse is resistant to both EAE and collagen induced arthritis (23,24).

Th17 cells cause inflammation by tumor necrosis factor a (TNF-α), IL-17A, IL-17F, IL-21 and IL- 22, leading to up-regulated expression of numerous pro-inflammatory cytokines and chemokines such as CXCL1, CXCL8, and CCL2 and IL-6, by local tissue and infiltrating inflammatory cells. These inflammatory mediators may further increase the recruitment of different leukocyte subsets, ultimately leading to target organ injury. Interleukin-17 was isolated and characterized 10 years before identification of the Th17 lineage from a rodent CD4^+^ T-cell cDNA library (25). It represents the founder member of a cytokine family which includes IL-17, IL-17B, IL-17C, IL-17D, IL-17E, IL-17F and the receptors IL-17RA, IL-17RB, IL-17RC, IL-17RD, and IL-17RE (26). The biologically most potent member of the family is IL-17A that binds to the IL-17RA and IL-17RC receptors.

The main function of the Th17 immune response is in the defense against fungal and bacterial pathogens (27). The presence of IL-17-producing T cells in biopsies of patients with multiple sclerosis, rheumatoid arthritis and Crohn’s disease, suggests that the Th17 immune response may also contributes to tissue injury in human autoimmune and inflammatory diseases (28). However, at the site of inflammation, the numbers of IL-17-producing T cells are often low and they usually are found together with a more prominent infiltrate of Th1 cells. This may suggest that the combined action of pathogenic Th1 and Th17 cells might be responsible for establishment of autoimmunity in humans.

### 3.2. Correlation between Th17 cells and Treg and Th1 cells

The polarization of naive CD4^+^ Th cells into Th17 and Treg cells requires TGF-β. Unopposed TGF-β stimulation in the context of antigen presentation induces Foxp3 expression and immunoregulation Treg commitment. However, in the context of inflammation signaled by presence of IL-6, TGF-β drives inflammation and Th17 differentiation (29). Furthermore, IL-6 inhibits the differentiation of Tregs and generation of Foxp3. It facilitates Th17 effector cells by reducing the functional capacity of Tregs, too (30). These observations may suggest a relationship between Th17 and Tregs differentiation depending on the presence of inflammatory danger signal IL-6 (31). Interleukin-21, the Th17-amplifying cytokine, inhibits TGF-β induction of Tregs (32,33). Interleukin-27, an IL-2 family cytokine, is a negative regulator of Th17 development (34). Interleukin-2 also induces Tr1 cells, a Treg population characterized by IL-10 expression (35). Interleukin-10 is also a negative regulator of Th17 development (36). Interleukin-17A receptor is expressed on differentiated Th1 cells. *In vitro* experiments show that IL-17A is able to down-regulate expression of the Th1 transcription factor, T-bet (37). These may suggest that Th17 cells are able to regulate pathogenic Th1- mediated tissue inflammation (38). In the presence of IL-12 or IL- 23 and absence of TGF-β, differentiated Th17 cells lose IL-17A and IL-17F secretion and become IFN-γ-producing cells (39). However, this switch is dependent on the Th1 transcriptions factors, STAT4 and T-bet, and show that Th17 cells might be not terminally differentiated but are capable of substantial plasticity.

### 3.3. Direct evidence implicating Th1 and Th17 cells in the pathogenesis of proliferative glomerulonephritis

Direct evidence that Th1 and Th17 cells may induce proliferative glomerulonephritis has been reported, where ovalbumin is planted in glomeruli of Rag1^-/-^ mice which are deficient in T and B cells. Injection of either ovalbumin-specific Th1 or Th17 polarized cells will induce proliferative glomerulonephritis (40-42). Th17 cell induced injury has been shown to develop early however, Th1 cell-mediated glomerulonephritis is more delayed and featured enhanced macrophage activation. These findings may support a model in which some forms of proliferative glomerulonephritis may be Th17 cell predominant, while others are Th1 mediated. It may also suggest that Th17 cells might dominate glomerular diseases that are neutrophil rich. However, other recent evidence in autoimmune renal disease suggests a role for Th17 in macrophage recruitment (43).

### 3.4. Th17 cells in anti-GBM glomerulonephritis

Anti-GBM glomerulonephritis has been suggested to be Th1 mediated due to the predominance of the Th1-associated IgG antibody subclasses (IgG1 and/or IgG3) and the presence of delayed-type hypersensitivity (DTH) effectors deposited in the kidney (41). It should be noted that the presence of DTH effectors may not be solely reliant on a Th1-mediated response as human trans-vivo DTH experiments that cell mediated autoimmunity against collagen (V) is dependent on IL-17, but not on IFN-γ (44). Moreover, the DTH response is diminished upon depletion of ether one of the human Th17- inducing cytokines (TGF-β or IL-1β), or CD4^+^ cells suggesting that Th17-mediated responses alone are able to mediate the DTH-like glomerular effects seen in patients with crescentic GN (44). Experimental autoimmune anti-GBM studies have shown that animals deficient in IFN-γ are not protected from disease and may develop more severe signs of clinical disease (45,46).

It has also been shown that when IL-23p19-deficient (IL-12 intact, IL-23 deficient) and IL-12p40 (IL- 12 and IL-23 deficient) mice are protected from the induction of experimental autoimmune anti- GBM but IL-12p35-deficient (IL-12 deficient, IL- 23 intact) mice are not (46). In this experimental model, the autoimmunity was induced in mice by repeated immunization with mouse alpha 3 chain Type IV collagen non-collagenous domain (a3(IV) NC1), which is the known target autoantigen in human autoimmune anti-GBM GN disease and Goodpasture’s disease (41). These observations may suggest that IL-23 and hence the Th17 cell subset are necessary for the induction of autoimmune renal disease. This is consistent with other suggestions in autoimmune inflammatory models of rheumatoid arthritis (47) and multiple sclerosis (20) that have proven the IL-23-driven Th17 cell subset essential in autoimmune pathogenesis. Experimental models have also been used to study the role of Th17 cells in GN. In a study where, sheep antimouse GBM antibodies were used to induce GN, it showed that IL-17A- and IL-23p19-deficient mice were protected from glomerular injury (48). IL-17A upregulated the expression of pro-inflammatory chemokines: CCL2, CCL3 and CCL20 in mice mesangial cells in vitro (48). In separate experiments using this model, it has also been shown that Th17 cells use the chemokine receptor CCR6 (which binds to CCL20) to migrate into the kidney (49).

### 3.5. Pauci-immune ANCA-associated glomerulonephritis

Despite the paucity of immunoglobulin deposition in the glomeruli, this form of crescentic glomerulonephritis is correlated with circulating anti-neutrophil cytoplasmic antibodies (ANCA), which are largely specific for two neutrophil constituents, proteinase-3 or myeloperoxidase (MPO). There is evidence suggesting a crucial role for ANCA in pauci-immune crescentic glomerulonephritis. Cellular and humoral MPO-specific immune responses have been demonstrated to promote crescentic glomerulonephritis. Furthermore, MPO has been shown in these glomerular lesions as a planted antigen (50-52). Percentages of IL-17A-producing activated T cells is increased in ANCA-positive Wegener’s granulomatosis patients (53). Peripheral blood mononuclear cells (PBMCs) from patients with active Churg–Strauss syndrome demonstrated a higher IL- 17A production than normal controls (54). Higher levels of serum IL-17A, IL-23, MPO and Pr3-specific Th17 cells are present in subjects with ANCA-associated vasculitis (55). MPO-ANCA triggers the production of IL-6, IL-17A and IL-23 and increases the activation of neutrophils, conditions that promote Th17-mediated autoimmunity (56). The role of IL- 17A has been examined using IL-17A-deficient mice in anti-MPO glomerulonephritis. The mice lacking IL-17A were protected from disease, and IL-17A promoted neutrophil recruitment to glomeruli (43). In addition to its effects on neutrophils, IL-17A has been shown to promote macrophage recruitment in a neutrophil-dependent manner.

## 4. Conclusions

Identification of the Th17 subgroup has explained seemingly paradoxical observations and improved our understanding of immune-mediated inflammatory responses. Secretion of IL-17A, as well as IL-17F, IL- 21, IL-22, suggests that Th17 subset may play a crucial role as a pleiotropic pro-inflammatory Th subset. There is experimental evidence to support the notion that Th1 and Th17 cells contribute to kidney injury in renal inflammatory diseases like glomerulonephritis. Studies have suggested that Th17 cells are not differentiated cells and are capable of switching to a Th1 phenotype (39). It has been hypothesized that following its differentiation and expansion by IL-6, TGF-β, IL-21 and IL-23, the Th17 can be recruited to the kidney and mediate tissue damage by mobilizing and activating neutrophils, planting neutrophil chemoattractants in the target organ, which in turn cause injury to the target tissue.

## Conflict of interests

The authors declare no conflict of interest.

## Authors’ contributions

All authors contributed to the manuscript equally.

## Funding/Support

None.
